# The Fer tyrosine kinase regulates interactions of Rho GDP-Dissociation Inhibitor α with the small GTPase Rac

**DOI:** 10.1186/1471-2091-11-48

**Published:** 2010-12-01

**Authors:** Fei Fei, Soo-Mi Kweon, Leena Haataja, Paulo De Sepulveda, John Groffen, Nora Heisterkamp

**Affiliations:** 1 Section of Molecular Carcinogenesis, Division of Hematology/Oncology, The Saban Research Institute of Childrens Hospital Los Angeles, CA 90027, USA; 2 Leukemia Research Program, Childrens Hospital Los Angeles, CA 90027, USA; 3 Metabolism, Endocrinology & Diabetes, University of Michigan, Ann Arbor, MI 48105, USA; 4 INSERM, UMR 891, Centre de Recherche en Cancérologie de Marseille, Laboratoire de Signalisation, Hématopoïèse et Mécanismes de l'Oncogenèse, Marseille, France; 5 Department of Pathology, Keck School of Medicine, University of Southern California, Los Angeles, CA 90033, USA

## Abstract

**Background:**

RhoGDI proteins are important regulators of the small GTPase Rac, because they shuttle Rac from the cytoplasm to membranes and also protect Rac from activation, deactivation and degradation. How the binding and release of Rac from RhoGDI is regulated is not precisely understood.

**Results:**

We report that the non-receptor tyrosine kinase Fer is able to phosphorylate RhoGDIα and form a direct protein complex with it. This interaction is mediated by the C-terminal end of RhoGDIα. Activation of Fer by reactive oxygen species caused increased phosphorylation of RhoGDIα and pervanadate treatment further augmented this. Tyrosine phosphorylation of RhoGDIα by Fer prevented subsequent binding of Rac to RhoGDIα, but once a RhoGDIα-Rac complex was formed, the Fer kinase was not able to cause Rac release through tyrosine phosphorylation of preformed RhoGDIα-Rac complexes.

**Conclusions:**

These results identify tyrosine phosphorylation of RhoGDIα by Fer as a mechanism to regulate binding of RhoGDIα to Rac.

## Background

Rac proteins belong to the larger family of Rho GTPases that act as molecular switches. When bound to GTP, they are in an active conformation. Hydrolysis of GTP to GDP causes an inactivating conformational change. Racs are post-translationally modified by the removal of C-terminal amino acid residues, followed by the attachment of a lipid moiety in the form of a geranyl-geranyl group [1].

Because of this modification, mature Rac proteins are lipophilic and are only present in the cytosol when bound to the chaperone and transport protein RhoGDI. RhoGDI regulates the movement of Rac between the cytosol and membranes. Moreover, a recent study showed that Rho proteins compete for binding to a limited pool of RhoGDI, which, once it has bound them, provides stabilization and protection from degradation [2].

There are only three distinct RhoGDI proteins, of which RhoGDIα is the most ubiquitously expressed. The structure of RhoGDI bound to small GTPases has been elucidated. Rac makes contact with both N-and C-terminal regions in RhoGDI, with its hydrophobic tail fitting into a pocket in the C-terminal part of RhoGDI. However, the exact mechanism by which binding and release of Racs from RhoGDI is mediated is not well known [3,4].

RhoGDI can be phosphorylated on serine S101 and S174 by the serine/threonine kinase Pak [5] and PKCα also phosphorylates RhoGDIα, on residue S96 [6]. Interestingly, constitutively activated oncogenic Src, which has a deregulated tyrosine kinase activity, was shown to phosphorylate RhoGDIα on tyrosine residue Y156 [7]. In the current study we report that a normal cellular tyrosine kinase which is member of the Src family, Fer, is able to specifically tyrosine phosphorylate RhoGDIα and that this can regulate the binding to Rac1.

## Results and discussion

**Figure 1:**
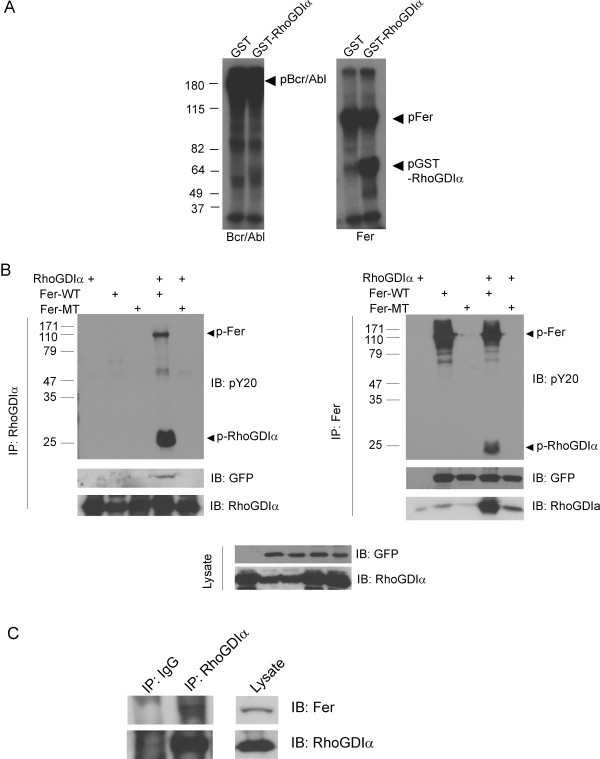
**Fer forms a complex with RhoGDIα and phosphorylates it on tyrosine**. (A) COS-1 cells were transfected with Bcr/Abl or Fer (pSG5-Fer). Autoradiogram of immune complex kinase reaction performed with immunoprecipitated Bcr/Abl or Fer (CH-6 antibodies) as indicated, with bacterially expressed GST-RhoGDIα or control GST added. (B) Immunoprecipitation (IP) from cells transfected with pEGFP tagged-Fer-WT or Fer-MT alone or together with RhoGDIα using RhoGDIα antibodies (Santa Cruz) (left panel) or Fer monoclonal rat antiserum (right panel). Antibodies used for immunoblot (IB) are indicated below the panels. After reaction with the pY20 antibodies, blots were stripped and reacted with RhoGDIα (Santa Cruz) or GFP antibodies. Arrowheads point to the locations of Fer and RhoGDIα. Molecular weight standards are in kDa. (C) Co-immunoprecipitation of endogenous Fer (using antibodies from Abcam) and RhoGDIα (Santa Cruz antibodies) proteins in RBL-2H3 mast cell lysates. These results are representative of three independent experiments.

The *BCR/ABL *oncogene encodes a chimeric protein that contains most of the Abl tyrosine kinase. Proteomic analysis of cells transfected with Bcr/Abl [8] reported changes in phosphorylation of RhoGDI. To investigate if the deregulated Bcr/Abl tyrosine kinase, similar to oncogenic Src [7], is able to modify RhoGDIα, we performed an immunocomplex kinase assay with cells expressing Bcr/Abl or with Fer, a non-receptor tyrosine kinase related to Abl, in the presence of added recombinant GST-RhoGDIα or control GST. As shown in Figure 1A, left panel, the Bcr/Abl protein exhibited strong autophosphorylation activity, as expected. However, there was very little phosphorylated RhoGDIα. In contrast, there was a prominent signal of phosphorylated RhoGDIα in the sample expressing Fer (Figure 1A right panel).

To examine whether Fer and RhoGDIα interact in cells, we transfected COS-1 cells with pEGFP-Fer (wild type, WT) or kinase dead D742R mutant pEGFP-Fer (mutant, MT), with or without RhoGDIα and investigated possible protein complex formation between these by co-immunoprecipitation. As shown in Figure 1B, left panel, Fer-WT clearly co-immunoprecipitated with RhoGDIα, which was phosphorylated on tyrosine, as judged by Western blotting using phosphotyrosine antibodies. In the converse experiment (Figure 1B, right panel, Fer IP) we confirmed the interaction between Fer-WT and RhoGDIα. In the presence of the Fer mutant lacking tyrosine kinase activity, no tyrosine phosphorylated RhoGDIα was detected, indicating that Fer tyrosine kinase activity is directly or indirectly needed to generate tyrosine phosphorylated RhoGDIα. Interestingly, the kinase-defective Fer protein exhibited a reduced ability to form a complex with RhoGDIα (compare lanes in IP Fer, IB for RhoGDIα), suggesting that the binding of these two proteins is facilitated by tyrosine phosphorylation. This is in agreement with the finding that the isolated Fer SH2 domain binds to RhoGDIα on far-Western blots (not shown). Importantly, we also were able to co-immunoprecipitate endogenous Fer with endogenous RhoGDIα in a lysate of the mast cell line RBL-2H3. This indicates that the complex formation between RhoGDIα and Fer also takes place without overexpression (Figure 1C).

**Figure 2:**
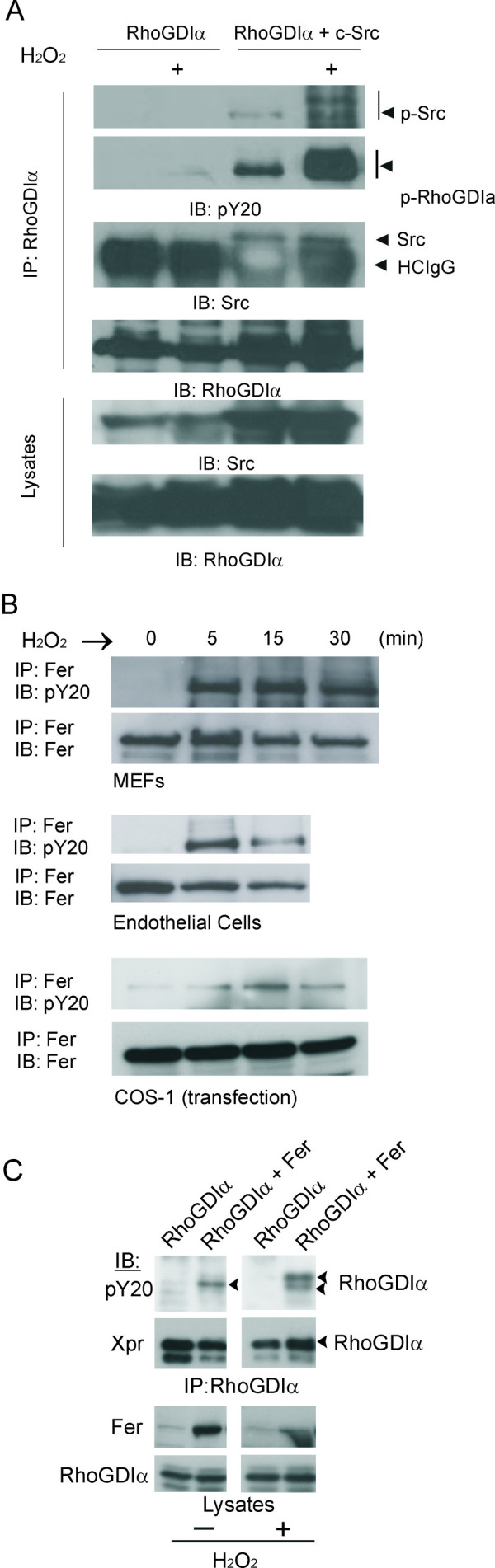
**Activation of Fer and non-oncogenic Src**. (A) RhoGDIα was expressed alone or with c-Src as indicated above the panel. Cells in lanes marked with a "+" were stimulated with H_2_O_2 _(10 mM) for 15 minutes and RhoGDIα was immunoprecipitated. Anti-phosphotyrosine (pY20) blots were stripped and re-probed with RhoGDIα antibodies (Santa Cruz). (B) Mouse embryonic fibroblasts (MEFs), mouse endothelial cells and cells transfected with Fer were treated with 5 mM of H_2_O_2 _for the indicated time. Fer protein was immunoprecipitated from the lysates followed by a pY20 immunoblot to detect activated tyrosine phosphorylated Fer (Abcam). (C) COS-1 cells transfected with Xpress-tagged RhoGDIα were stimulated or not with H_2_O_2_, as indicated below the panels. RhoGDIα antibodies (Upstate Biotechnology) were used for immunoprecipitation. Antibodies used for Western blotting are indicated to the left of the panels. Fer, CH-6 antibodies. This result is representative of two independent experiments.

Although reactive oxygen species (ROS) were originally identified as a bactericidal mechanism, a role for H_2_O_2 _as secondary messenger has now been well-established. Many groups suggest that Src may be a primary target of ROS, with some studies reporting activation of Src kinase activity upon ROS exposure [9]. Since constitutively activated oncogenic Src had been shown to phosphorylate RhoGDIα [10,11], we tested if exposure of c-Src to H_2_O_2 _would activate it to phosphorylate RhoGDIα. As shown in Figure 2A, in the presence of c-Src and after H_2_O_2 _exposure, we could detect tyrosine phosphorylated RhoGDIα. Sangrar et al [12] showed that ROS also induce Fer tyrosine autophosphorylation as well as trans-phosphorylation activity. As shown in Figure 2B, we confirmed Fer activation by H_2_O_2 _in different types of cells by immunoprecipitation and Western blotting with anti-phosphotyrosine antibodies. In addition, when we co-transfected Fer with RhoGDIα and treated the cells with H_2_O_2_, increased RhoGDIα tyrosine phosphorylation was detected (Figure 2C). The tyrosine phosphorylated RhoGDIα was visible as two discrete bands, indicating this protein is subject to processive phosphorylation.

**Figure 3:**
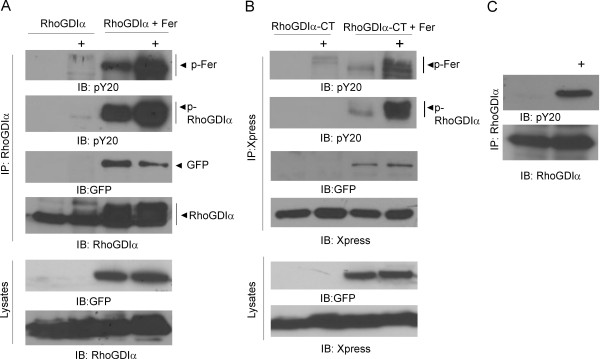
**C-terminal end of RhoGDIα is tyrosine phosphorylated**. (A) RhoGDIα was expressed alone or with pEGFP-tagged Fer as indicated above the panel. Cells were stimulated with pervanadate (0.5 mM H_2_O_2 _+ 0.5 mM vanadate) (+) and RhoGDIα was immunoprecipitated. pY20 blots were stripped and re-probed with GFP or RhoGDIα antibodies. Antibodies used for detection are indicated below the panels. (B) Xpress-tagged C-terminal RhoGDIα (CT) was expressed alone or together with pEGFP-tagged Fer. Cells were stimulated with pervanadate for 5 minutes. Results are representative of three independent experiments. (C). RBL-2H3 cells were treated with pervanadate for 5 minutes (+) as indicated and endogenous RhoGDIα was immunoprecipitated. Western blots were reacted with antibodies against RhoGDIα or phosphotyrosine. The RhoGDIα antibody was from Santa Cruz.

Tyrosine phosphorylation of signal transduction proteins in the absence of an oncogenic, constitutively activated tyrosine kinase may be difficult to detect due to the transient nature of the phosphorylation and the activity of phosphatases. Wang et al. reported that pervanadate treatment allows detection of phosphorylation on RhoGDIα because this inhibits protein-tyrosine phosphatases and traps proteins in their tyrosine-phosphorylated form [13]. Next, we examined if pervanadate would further activate Fer and increase RhoGDIα tyrosine phosphorylation. As shown in Figure 3A, when cells co-expressing RhoGDIα and Fer were exposed to pervanadate for 5 minutes, a marked increase in tyrosine phosphorylation was seen both in Fer and RhoGDIα proteins (Figure 3A). The N-terminal regulatory domain of RhoGDI binds to the switch region of Rac whereas the C-terminal end adopts a conformation that accommodates the hydrophobic tail of Rac [6]. To examine which domain interacts with Fer, we expressed the N- and C-terminus of RhoGDIα as Xpress-tagged proteins, together with Fer, and immunoprecipitated each fragment of RhoGDIα with Xpress antibodies. We could not detect a clear interaction of the N-terminal end with Fer (data not shown). As shown in Figure 3B (RhoGDIα-CT + Fer), in immunoprecipitates with the C-terminal end of RhoGDIα and in the presence of pervanadate, we recovered tyrosine-phosphorylated Fer. Moreover, this domain of RhoGDIα also was tyrosine phosphorylated (Figure 3B). This indicates that Fer interacts with and tyrosine phosphorylates residues in the part of RhoGDIα that includes the domain that binds to the C-terminal end of Rac. We were also able to demonstrate that endogenous RhoGDIα was phosphorylated on tyrosine in response to pervanadate, which activates Fer in RBL-2H3 cells (Figure 3C).

**Figure 4:**
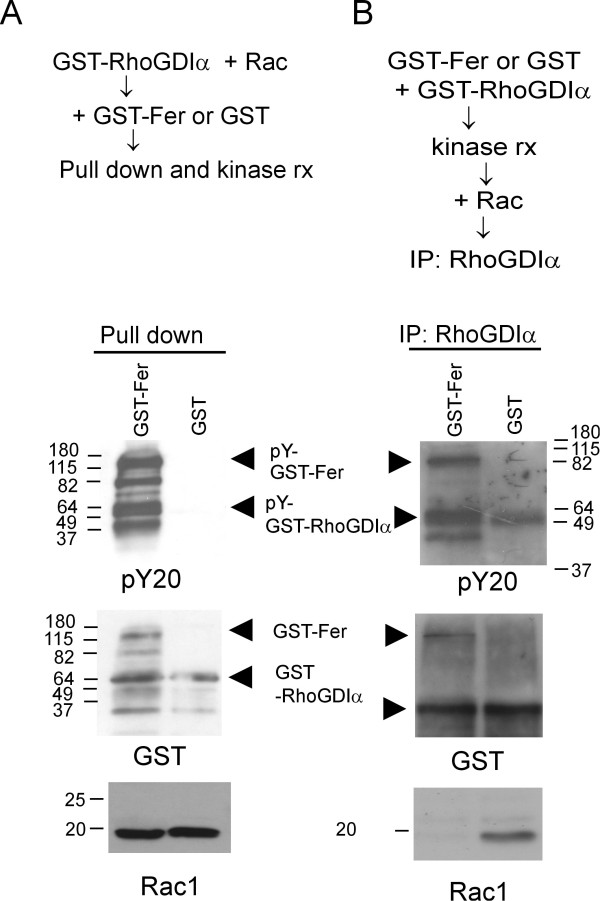
**Fer interferes with binding of RhoGDIα and Rac**. (A) GST-RhoGDIα was pre-incubated with Rac, after which GST-Fer (G-Fer) or GST (G) was added and GST fusion proteins were pulled down with glutathione agarose, followed by kinase reactions. (B) GST-RhoGDIα was pre-incubated with GST-Fer or GST in kinase buffer, after which Rac was added and RhoGDIα was immunoprecipitated. 50 pmol of RhoGDIα and Rac1, with 25 pmol of GST and GST-Fer were used. Blots were reacted with Rac1 antibodies or pY20, then stripped and reacted with GST antibodies. This result is representative of two independent experiments.

We used purified proteins to examine if tyrosine phosphorylation of RhoGDIα could regulate its ability to bind Rac. Fer was expressed as a GST fusion protein in baculovirus. In Figure 4A (left panel), we first incubated GST-RhoGDIα with Rac to allow these proteins to form a complex. Then GST-Fer or control GST was added with a kinase reaction buffer and the kinase reaction was initiated. As shown in the top panel (pY20 blot), both autophosphorylation of Fer and transphosphorylation of RhoGDIα occurred as expected. However, there was no evidence that Rac was tyrosine phosphorylated (not shown). Rac protein was recovered in immunoprecipitates from both Fer-treated and non-Fer treated samples, indicating that the presence or absence of Fer does not affect preformed Rac-RhoGDIα complexes. A different result was obtained when Fer was first incubated with RhoGDIα and a kinase reaction was done. When Rac was subsequently added and allowed to bind, we found that it was not co-immunoprecipitated with RhoGDIα in the Fer-treated sample (Figure 4B). The lack of binding of Rac to RhoGDIα could be because tyrosine-phosphorylated RhoGDIα has decreased affinity for Rac, or because the binding of Fer to RhoGDIα prevented subsequent binding of Rac to RhoGDIα. Interestingly, this effect is similar to that reported for oncogenic Src: when Src phosphorylated RhoGDIα, the affinity of RhoGDIα for Rac was also significantly reduced [7].

## Conclusions

Tyrosine phosphorylation as a mechanism to regulate RhoGDIα has not been widely investigated, although it was recently reported that pervanadate treatment of a pancreatic islet beta cell line caused the appearance of phosphotyrosine on RhoGDIα and was accompanied by lack of Cdc42 binding [13]. There is also increasing evidence that H_2_O_2 _regulates the balance of tyrosine phosphorylation and de-phosphorylation in cells. The current study shows that non-oncogenic tyrosine kinases are likely to play a role in regulating the binding of Rac GTPases to RhoGDIα and through this, the cellular pool of Rac.

## Methods

### Antisera and DNA constructs

The Fer polyclonal rabbit anti-Fer CH-6 antiserum [14,15] and anti-Fer monoclonal rat antiserum [16] have been previously described and were used for immunoprecipitation. RhoGDIα antibodies were obtained from Upstate Biotechnology (Temecula, CA) or Santa Cruz (Santa Cruz, CA). Antibodies against Fer, GFP, Xpress and GST were from Abcam (San Francisco, CA), Santa Cruz, Invitrogen (Carlsbad, CA) and Santa Cruz, respectively. Antibodies against pY20 and Rac1 were purchased from BD Bioscience (Franklin Lakes, NJ). pSG5-Fer was constructed by subcloning a 2.9 kb BamHI fragment from human Fer clone L3, encompassing the entire coding region and small 5' and 3' untranslated regions, into the BamHI site of pSG5. pEGFP-Fer (WT), pEGFP-Fer (MT D742R) and RhoGDIα have been previously described [17,18]. The N- and C-terminal fragments of RhoGDIα were subcloned from pEGFP-RhoGDIα-NT and pEGFP-RhoGDIα-CT into pcDNA3.1/HisB by digestion with EcoRI/XbaI and have been previously described [18]. p-LNCX mouse c-Src was obtained from Addgene (Cambridge, MA).

### Recombinant proteins

A fragment containing the start codon of human Fer (5'-ggggatccatggggtttgggagtgacctg) was used to introduce a BamHI site immediately 5' to the Fer ATG. A 155 bp fragment amplified with this primer and a 3' oligonucleotide was digested with BamHI × NsiI and the 5' BamHI-NsiI fragment ligated with a 3' 1.2 kb NsiI-KpnI fragment isolated from plasmid Fer SV-7-2 [19] into pSK digested with BamHI × KpnI. A 1.4 kb BamHI-KpnI fragment from this plasmid was ligated with a 1.3 kb KpnI-EcoRI fragment into the pAcG2T baculovirus transfer vector digested with BamHI × EcoRI. *S. frugiperda *Sf9 cells were grown in Grace's Insect medium (Invitrogen) with 10% FBS (heat-inactivated) and 10 μg/ml Gentamycin (Invitrogen). DNA transfections used 0.5 μg BaculoGold viral DNA and 2 μg of recombinant baculovirus transfer vector. Three rounds of viral amplification were performed and the third amplification used for large-scale infection and baculovirus production. GST fusion proteins were purified by glutathione-agarose. GST-Fer was washed and concentrated using a Centricon-10 (Amicon) filter. Bacterially expressed RhoGDIα and Rac1 were prepared as previously described, with the GST tag removed from the Rac1 protein by thrombin cleavage [18].

### Cell lines and immunoprecipitations

COS-1 cells and rat basophilic leukemia/mast cell RBL-2H3 cells were obtained from the ATCC (Rockville, MD). All tissue culture media and supplements were from Invitrogen (Carlsbad, CA). COS-1 cells were cultured in DMEM or RPMI containing 10% fetal bovine serum (FBS), penicillin (100 U/ml), streptomycin (100 μg/ml), L-glutamine and sodium pyruvate (1 mM). RBL-2H3 cells were cultured in MEM containing 15% FBS, 1% penicillin/streptomycin. Immunoprecipitations and immunoblotting were performed as described before [18].

### Immunocomplex kinase assay

The kinase activity of Fer or Bcr/Abl was measured as described previously [18]. Briefly, Fer-transfected COS-1 cells were lysed with 1× RIPA buffer (50 mM Tris-HCl, pH 8.0, 150 mM NaCl, 1% Igepal, 0.5% deoxycholate, 0.1% SDS, 5 mM EDTA) containing PMSF, leupeptin, and aprotinin and then incubated with Fer antibody, CH-6 for 2 h at 4°C. After incubation with protein A agarose for 30 min at 4°C, the affinity complex was washed with ice-cold high salt HNTG buffer (80 mM HEPES, pH 7.5, 0.1% Triton-X100, 500 mM NaCl and 10% glycerol) and then with ice-cold low salt HNTG buffer (80 mM HEPES, pH 7.5, 0.1% Triton-X100, 150 mM NaCl and 10% glycerol). For kinase reactions, 10 μl of 40 mM PIPES and 10 μl of 40 mM MnCl_2 _were added as well as 0.5 μg of GST-RhoGDIα protein or GST protein. The reactions were initiated by addition of 10 μCi of [γ^32^P]ATP (5000 Ci/mmol) (Amersham). After 40 min on ice, reactions were terminated by the addition of 2× SDS-sample buffer and boiling for 5 min. Samples were run on 8% SDS-PAGE gels and the radioactively labeled proteins visualized by autoradiography.

### *In vitro* binding assay for interaction of RhoGDIα and Rac1

To investigate possible binding of phosphorylated-RhoGDIα to Rac1, GST-RhoGDIα protein (50 pmol) was first combined with GST-Fer (25 pmol) or GST alone (25 pmol) in 100 μl of kinase buffer (20 mM HEPES, pH 7.5, 50 mM NaCl, 25 mM MnCl_2_, 10 mM MgCl_2_, 1 mM DTT). 20 μM of ATP was added to the mixture, which was incubated for 30 min at 4°C for the kinase reaction. Next, 400 μl of binding buffer (1× DPBS, 0.1% IgePal, 10% glycerol, 0.5 mM DTT containing the protease inhibitors PMSF, leupeptin, and aprotinin) and 50 pmol Rac1 protein were added, followed by incubation for 1 h at 4°C. Incubation with antibody against RhoGDIα for 1 h at 4°C was followed by Protein A agarose addition and a second incubation 1 h at 4°C. To investigate if phosphorylation of RhoGDIα by Fer releases Rac1, GST-RhoGDIα protein was incubated with Rac1 in binding buffer for 1 h at 4°C; GST-Fer or GST alone was added, and a pull down with glutathione agarose was done, followed by washing of the precipitate. Kinase reactions were initiated by addition of kinase buffer and ATP for 30 min at 4°C.

## List of abbreviations

RhoGDI: Rho GDP-dissociation inhibitor; ROS: reactive oxygen species

## Authors' contributions

FF performed experiments, brought in new ideas and wrote part of the manuscript; S-M K performed experiments and wrote part of the manuscript; LH made the baculovirus Fer protein; PS provided critical reagents; JG designed experiments and provided critical input; NH provided ideas and wrote the manuscript. All authors read and approved the final manuscript.

## References

[B1] Ridley AJ Rho GTPases and actin dynamics in membrane protrusions and vesicle trafficking Trends Cell Biol 2006 16 522 529 10.1016/j.tcb.2006.08.006 16949823

[B2] Boulter E Garcia-Mata R Guilluy C Dubash A Rossi G Brennwald PJ Burridge K Regulation of Rho GTPase crosstalk, degradation and activity by RhoGDI1 Nat Cell Biol 2010 12 477 483 10.1038/ncb2049 20400958 PMC2866742

[B3] Dovas A Couchman JR RhoGDI: multiple functions in the regulation of Rho family GTPase activities Biochem J 2005 390 Pt 1 1 9 16083425 10.1042/BJ20050104 PMC1184558

[B4] Dransart E Olofsson B Cherfils J RhoGDIs revisited: novel roles in Rho regulation Traffic 2005 6 957 966 10.1111/j.1600-0854.2005.00335.x 16190977

[B5] DerMardirossian C Schnelzer A Bokoch GM Phosphorylation of RhoGDI by Pak1 mediates dissociation of Rac GTPase Mol Cell 2004 15 117 127 10.1016/j.molcel.2004.05.019 15225553

[B6] Knezevic N Roy A Timblin B Konstantoulaki M Sharma T Malik AB Mehta D GDI-1 phosphorylation switch at serine 96 induces RhoA activation and increased endothelial permeability Mol Cell Biol 2007 27 6323 6333 10.1128/MCB.00523-07 17636025 PMC2099605

[B7] DerMardirossian C Rocklin G Seo JY Bokoch GM Phosphorylation of RhoGDI by Src regulates Rho GTPase binding and cytosol-membrane cycling Mol Biol Cell 2006 17 4760 4768 10.1091/mbc.E06-06-0533 16943322 PMC1635405

[B8] Unwin RD Sternberg DW Lu Y Pierce A Gilliland DG Whetton AD Global effects of BCR/ABL and TEL/PDGFRbeta expression on the proteome and phosphoproteome: identification of the Rho pathway as a target of BCR/ABL J Biol Chem 2005 280 6316 6326 10.1074/jbc.M410598200 15569670

[B9] Sun G Kemble DJ To C or not to C: direct and indirect redox regulation of Src protein tyrosine kinase Cell Cycle 2009 8 2353 2355 19571676 10.4161/cc.8.15.9225

[B10] Mehdi MZ Pandey NR Pandey SK Srivastava AK H2O2-induced phosphorylation of ERK1/2 and PKB requires tyrosine kinase activity of insulin receptor and c-Src Antioxid Redox Signal 2005 7 1014 1020 10.1089/ars.2005.7.1014 15998256

[B11] Rosado JA Redondo PC Salido GM Gomez-Arteta E Sage SO Pariente JA Hydrogen peroxide generation induces pp60src activation in human platelets: evidence for the involvement of this pathway in store-mediated calcium entry J Biol Chem 2004 279 1665 1675 10.1074/jbc.M307963200 14581479

[B12] Sangrar W Gao Y Scott M Truesdell P Greer PA Fer-mediated cortactin phosphorylation is associated with efficient fibroblast migration and is dependent on reactive oxygen species generation during integrin-mediated cell adhesion Mol Cell Biol 2007 27 6140 6152 10.1128/MCB.01744-06 17606629 PMC1952165

[B13] Wang Z Thurmond DC Differential phosphorylation of RhoGDI mediates the distinct cycling of Cdc42 and Rac1 to regulate second-phase insulin secretion J Biol Chem 2010 285 6186 6197 10.1074/jbc.M109.072421 20028975 PMC2825414

[B14] Hao QL Ferris DK White G Heisterkamp N Groffen J Nuclear and cytoplasmic location of the FER tyrosine kinase Mol Cell Biol 1991 11 1180 1183 1990274 10.1128/mcb.11.2.1180-1183.1991 PMC359807

[B15] Rosato R Veltmaat JM Groffen J Heisterkamp N Involvement of the tyrosine kinase fer in cell adhesion Mol Cell Biol 1998 18 5762 5770 9742093 10.1128/mcb.18.10.5762 PMC109162

[B16] Voisset E Lopez S Chaix A Georges C Hanssens K Prebet T Dubreuil P De Sepulveda P FES kinases are required for oncogenic FLT3 signaling Leukemia 2010 24 721 728 10.1038/leu.2009.301 20111072

[B17] Itoh T Hasegawa J Tsujita K Kanaho Y Takenawa T The tyrosine kinase Fer is a downstream target of the PLD-PA pathway that regulates cell migration Sci Signal 2009 2 ra52 10.1126/scisignal.2000393 19738202

[B18] Kweon SM Cho YJ Minoo P Groffen J Heisterkamp N Activity of the Bcr GTPase-activating domain is regulated through direct protein/protein interaction with the Rho guanine nucleotide dissociation inhibitor J Biol Chem 2008 283 3023 3030 10.1074/jbc.M705513200 18070886

[B19] Hao QL Heisterkamp N Groffen J Isolation and sequence analysis of a novel human tyrosine kinase gene Mol Cell Biol 1989 9 1587 1593 2725517 10.1128/mcb.9.4.1587-1593.1989 PMC362575

